# Knowledge transfer for the management of dementia: a cluster-randomised trial of blended learning in general practice

**DOI:** 10.1186/1748-5908-5-1

**Published:** 2010-01-04

**Authors:** Horst C Vollmar, Herbert Mayer, Thomas Ostermann, Martin E Butzlaff, John E Sandars, Stefan Wilm, Monika A Rieger

**Affiliations:** 1 Institute of General Practice and Family Medicine, Witten/Herdecke University, Witten, Germany; 2 Fraunhofer Institute for Systems and Innovation Transfer (ISI), Karlsruhe, Germany; 3 Institute for Research and Transfer in Dementia Care, Partner Site of the German Centre for Neurodegenerative Diseases, Helmholtz Association, Witten, Germany; 4 Department of Nursing Science, Witten/Herdecke University, Witten, Germany; 5 Chair of Medical Theory, Integrative and Anthroposophical Medicine, Witten/Herdecke University, Herdecke, Germany; 6 Medical Education Unit, The University of Leeds, Leeds, UK; 7 Institute of Occupational and Social Medicine, University and University Hospital, Tübingen, Germany

## Abstract

**Background:**

The implementation of new medical knowledge into general practice is a complex process. Blended learning may offer an effective and efficient educational intervention to reduce the knowledge-to-practice gap. The aim of this study was to compare knowledge acquisition about dementia management between a blended learning approach using online modules in addition to quality circles (QCs) and QCs alone.

**Methods:**

In this cluster-randomised trial with QCs as clusters and general practitioners (GPs) as participants, 389 GPs from 26 QCs in the western part of Germany were invited to participate. Data on the GPs' knowledge were obtained at three points in time by means of a questionnaire survey. Primary outcome was the knowledge gain before and after the interventions. A subgroup analysis of the users of the online modules was performed.

**Results:**

166 GPs were available for analysis and filled out a knowledge test at least two times. A significant increase of knowledge was found in both groups that indicated positive learning effects of both approaches. However, there was no significant difference between the groups. A subgroup analysis of the GPs who self-reported that they had actually used the online modules showed that they had a significant increase in their knowledge scores.

**Conclusion:**

A blended learning approach was not superior to a QCs approach for improving knowledge about dementia management. However, a subgroup of GPs who were motivated to actually use the online modules had a gain in knowledge.

**Trial registration:**

Current Controlled Trials ISRCTN36550981.

## Background

General practitioners (GPs) need effective ways to keep their knowledge and skills up to date. Evidence-based medical guidelines seem to be helpful in this respect, but often effectiveness of guidelines is low due to insufficient dissemination and implementation [1-4]. Studies have shown a small but positive influence of continuing medical education (CME), continuing professional development (CPD), and knowledge transfer/translation (KT) on physicians' knowledge, attitudes, skills, and competences [5,6]. Recently, it has been suggested that the application of new information technologies in CME, CPD, and particularly KT, can have a lasting impact on physicians' learning behaviour [7-9]. Only a few studies have demonstrated significant effects on knowledge and skills by the use of e-learning and blended learning approaches [10-13].

In the context of chronic diseases with high prevalence and/or a high burden of disease, such as diabetes, depression, or dementia, KT is essential. As a result of the demographic shift, dementia in particular is recognized as an increasing and worldwide problem [14-16]. Nevertheless, several studies have documented deficits in the detection and management of dementia as well as problems in the implementation of guidelines [17-22].

A study by Downs and colleagues investigated the innovative use of electronic decision support software and practice-based workshops for dementia care and noted that this educational approach seemed to be effective [23]. However, the authors later stated that the adherence of GPs to a dementia guideline was lower than expected [24].

Up to now, no previous studies of the use of e-learning or blended learning for the training of GPs on dementia management were identified. Blended learning combines e-learning with standard teaching methods and various teaching/learning media. Thus, learning content is conveyed face-to-face as well as via web-based training (WBT), CD-Rom, or print media [25-28].

We therefore decided to conduct a cluster-randomised trial to compare knowledge acquisition about dementia management between a blended learning approach using online modules in addition to quality circles (QCs) and QCs alone [25].

## Methods

The WIDA-trial (acronym of the German term: KT about dementia in general practice) was conducted in a setting of GPs QCs in urban and rural areas of the western part of Germany [25]. QCs are regular regional meetings of GPs to discuss clinical topics, guidelines, and other ways to improve the quality of care as well as new developments in politics and funding. The participation of German GPs in QCs is mandatory in order to be part of government-funded disease management programs (DMPs) or to be part of pilot projects with health insurance funds. QCs also provide an opportunity to obtain CME credit points, which have been mandatory for GPs in Germany since January 2004. More than 50 percent of all German GPs are now organized in QCs [29]. Attendance of QCs has also been shown to change prescription patterns in general practice [30].

In our study, QCs were recruited for participation either by letter or through personal telephone call to the responsible QC moderator. We contacted all available GP QCs within a radius of 50 kilometres around Witten/Herdecke University regardless of their speciality. We asked the moderators to allow us to visit their QCs and train the GPs in the diagnosis and therapy of dementia according to a dementia guideline produced by the German Society for General Practice and Family Medicine (DEGAM) [31].

### Participants

**Figure 1:**
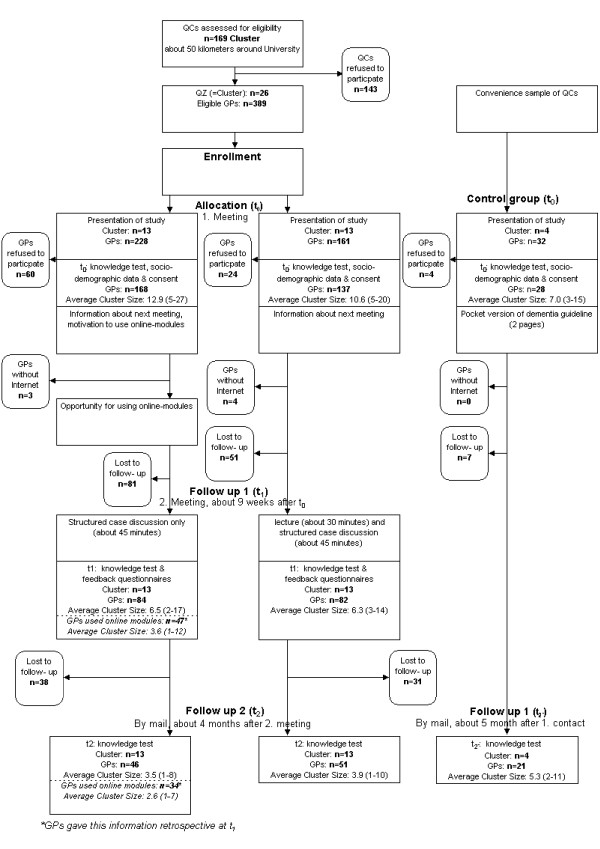
**Flow chart of the WIDA-trial**.

Members of the study team visited the QCs at their regular meeting places (*e.g*., surgery, restaurant, or other). After a short introduction to the study, the GPs were recruited and signed written consent was obtained. (t_0_, Figure 1). Recruited GPs were required to participate in an additional QC meeting (t_1_, Figure 1) and they were also required to have access to the internet [25]. The study participants received no reimbursement for participating in the WIDA-trial apart from CME credit points gained for attendance of the QC meetings and--in case of blended learning--for the online modules.

### Intervention

All GPs in one QC were randomised as a cluster to study arm A (blended learning--online modules and a structured discussion during a quality circle meeting) or study arm B (lecture and a structured discussion during a QC meeting). Participants in both study arms were asked to complete a 20-item knowledge test about dementia management before receiving an intervention (Additional File 1).

In both study arms, the intervention comprised the presentation of the guideline content with regard to diagnosis, management, and therapy of dementia either by blended learning or by face-to-face teaching. In both teaching forms, a structured case discussion was one of the teaching elements used during face-to-face teaching in the QC meeting. In study arm A, this case discussion was prepared by online modules to be completed before the QC meeting. In study arm B, the case discussion was prepared by a lecture given immediately before in the very QC meeting (the so-called 'classical approach').

### Study arm A

All participants were introduced to the online modules (t_0_, Figure 1) and were informed that a case discussion was scheduled for the next QC session (t_1_, Figure 1). Participants were expected to complete the online modules by independent learning before this next QC meeting. These online modules on the website included:

1. Two interactive case stories on dementia related to the guideline content (diagnosis or management and therapy of dementia).

2. Three testing modules allowing acquisition of CME credit points. They covered the same topics as the interactive case stories (as well as the lecture in study arm B).

3. The guideline was provided in two formats: html to click through the guideline and pdf for download.

4. The technical and educational details as well as the usability of the e-learning platform were reported elsewhere [32].

During the next QC meeting (t_1_), participants of study arm A immediately started with the structured case discussion (about 45 minutes, content identical to study arm B), there was no lecture as there was in study arm B. At the end of the meeting, participants were asked to complete the knowledge test (Additional File 1) about dementia management and an evaluation form [33]. The usage or non-usage of the online modules was checked by an additional self-reported questionnaire.

### Study arm B

Participants were informed that a lecture and a case discussion were scheduled for the next QC session. During this QC meeting (t_1_, Figure 1), GPs received a dementia-related training based on a slide presentation that lasted about 30 minutes. After the lecture, a structured case discussion was held identical to study arm A (about 45 minutes). At the end of the meeting, participants filled out the knowledge test (Additional File 1) about dementia management and an evaluation form [33].

### Study arm A and B

All participants were asked to complete a further knowledge test about dementia management that was sent by post after six months as well as a feedback questionnaire (t_2_). After the second QC meeting, all participants received a printed pocket version (two pages) of the guideline. Apart from those and the CME credit points (see above) no other incentives were offered.

### Control group

Because there may be confounding effects during the study due to changes in health care, such as dementia awareness campaigns, a (not randomised) control group was addressed. Participants in this group received only a printed pocket version (two pages) of the dementia guideline (Figure 1). The participants were also informed that they would receive a knowledge test again a few months later (t_2'_, Figure 1). Data from the control group was gathered only at t_0 _and t_2' _(approximately five months after t_0_, Figure 1).

### The time that study took place

The study started in August 2006 with inclusion of the QCs. The last educational training took place in July 2007. The last questionnaires were sent out in December 2007. The database was closed in June 2008 and evaluation was completed in September 2008.

### Instruments: the knowledge test

Prior to this study, we developed a 20-item knowledge test about dementia management with 10 multiple choice (MC) questions about the diagnosis of dementia and 10 MC questions dealing with dementia management and therapy. We performed a pilot of the knowledge test in a QC of GPs cooperating with Witten/Herdecke University and not included in our study. This pilot test resulted in data on the level of difficulty of the test and on possible ceiling effects, the latter being important as we planned to use the same test three times [25]. After a few corrections we used the knowledge test to evaluate 132 GPs during the dementia management initiative in general medicine (IDA) [34,35].

### Outcome criteria

The primary outcome was the knowledge gain (KG) between the knowledge test before (t_0_, Figure 1) and after the intervention (t_1_, Figure 1), calculated as the difference KG (t_1_-t_0_). Secondary outcomes included a comparison of the knowledge gain of the two groups at t_2 _(calculated as the difference t_2_-t_0_) (Figure 1). We also performed subgroup analyses to compare the knowledge gain in study arm B with the one in colleagues from study arm A, who indicated whether or not they used the online modules ('per protocol').

### Statistics

The Chi-Square-test was used to analyse dichotomous and categorical variables. The first evaluation without adjusting for cluster was carried out as follows: differences between the cumulative values of the knowledge test at t_0 _and t_1 _(t_1_-t_0_) and t_0 _and t_2 _(t_2_-t_0_), respectively, were determined. The mean differences in each group were analysed by a t-test. Mean values and standard deviation of difference values were indicated. To take the clustering into account, we performed an additional analysis of covariance (ANCOVA) [36,37].

All GPs who completed the knowledge test at t_0 _and t_1 _were analysed, even those who eventually did not use the additional e-learning opportunities. Subgroup analyses were performed on those GPs who answered that they had used or not used the online modules. Two-sided p-values ≤ 0.05 were considered significant. All tests and models were fitted using SPSS 17.

### Arrangements for data oversight: Cluster randomisation

Cluster randomisation took place at QC level (two arms). Stratified randomisation was performed by a statistician separately for small and large QCs (definition for large QCs: 12 or more participating GPs as reported by the QC moderators) [25]. Group allocation was then placed in sealed opaque envelopes with consecutive numbering of each stratum. Members of the study team did not know whether a QC was randomised into group A or group B until they had opened the envelope in front of the participating GPs at t_0 _[25].

### Sample Size

Based on the results of another study on teaching physicians on dementia diagnosis and therapy using the same knowledge test, we assumed an effect size of 0.5 and a standard error of α = 5% (power = 80%) [25].

In the former study a significant knowledge gain of 4.0 ± 2.6 questions (confidence interval 3.6 to 4.5, p < 0.001) was identified. The comparison of two different training groups displayed a difference of mean values of 3.1 ± 2.1 (p < 0.001). In both cases, this resulted in an effect size of 1.5 (Cohen's d) [34,35]. However, an effect size of 1.5 appeared to be too optimistic. A study in an US hospital compared an online training with a classical face-to-face training and assumed an effect size of 0.75 [10]. Extensive investigation did not identify directly comparable research on the effects of a blended-learning concept that could have served as a basis for sample size calculation. Therefore the WIDA study conservatively assumed an assessed medium effect size of 0.5.

Based on these assumptions, the sample size was calculated with 128 GPs in total. This sample size should allow us to check whether the two training concepts differed by approximately 0.5 SD, which corresponded to about one (or more) correctly answered question in the knowledge test. We assumed an intra-cluster correlation coefficient (ICC) of 0.04 and an average cluster size of 10 (= median of GPs per QC) [25,38]. So, the design effect was calculated as 1.36. This resulted in a sample size of n = 128 × 1.36 = 174 GPs (87 GPs per group) [25].

## Results

Out of 169 consecutive QCs, 26 moderators (15.4%) agreed to participate at a cluster level (Figure 1). The reasons for non-participation of QCs (as mentioned by the QC moderators) were different focus of the QCs (specialised only on diabetes, complementary and alternative medicine (CAM), or other topics), difficulties with schedules or lack of time, a previous meeting on dementia management, or lack of interest in the topic. The 26 participating QCs were randomised at t_0 _to either study arm A ('blended learning', n = 13 cluster) or study arm B ('classical approach', n = 13 cluster). Consequently, all GPs in one cluster were in the same study group. After the introduction, 305 GPs completed the knowledge test and the baseline documentation and gave informed consent (t_0_, August 2006 to May 2007). One hundred and sixty-eight (55%) were assigned to study arm A, and 137 (45%) to study arm B. Three GPs in study arm A and four in study arm B were excluded because they did not have internet access (Figure 1).

One hundred and sixty-six GPs completed the second knowledge test at the end of the second meeting (t_1_, September 2006 to July 2007), 84 (50.6%) in study arm A, and 82 (49.4%) in study arm B.

Ninety-seven GPs completed the third knowledge test after a period of about six months (t_2_, March 2007 to November 2007), 46 (47.4%) in study arm A, and 51 (52.6%) in study arm B.

Flow chart and characteristics of QCs and GPs are shown in Figure 1 and Table 1, respectively, following the CONSORT statement extension to cluster-randomised trials [39].

**Table 1:** Characteristics of participating QCs (= cluster) and GPs (= participants)

	Characteristics	Study arm A ('blended')	Study arm B ('classical')	'Control' Group (not randomised)
**QCs**		13	13	4
	Sponsored by pharmaceutical industry	5	4	0
	Training in dementia topics during the last 12 months	2	1	1
	Meetings per year (median)	6.7	6.5	6.5
	Average time between t_0 _and t_1 _in weeks (SD) ('control' group: t_2_')	9.5 (± 3.7)	8.5 (± 4.4)	21 (± 4.0)
				
**GPs**	Participants (t_0 _and t_1_)	84	82	21 (t_0 _and t_2_')
	Average age of participants in years (SD)	51 (± 6.8)	50.7 (± 7.5)	49.3 (± 8.8)
	Percentage of females	29%	28%	43%
	Single doctor practices (versus group practice)	44%	51%	24%

There were no significant differences between participants in groups A or B with regard to sponsorship of the QCs; in study arm B, the percentage of single doctor practices was slightly higher than in study arm A (Table 1).

### Primary Outcome: Difference in knowledge gain (t_1_-t_0_)

Study group A (n = 84) and B (n = 82) did not show any statistically significant difference in knowledge gain within all 20 questions at t_1 _(3.67 versus 3.60 questions, mean difference: 0.07; CI: -0.84 to 0.98; p = 0.881; T = 0.15). Baseline knowledge score significantly predicted knowledge score after intervention (F(1;162.04) = 31.81; p < 0.001). A cluster analysis (ANCOVA model) with QCs as a random effect and the pre-test (t_0_) as covariate showed a comparable result (adjusted mean difference = -0.020; CI: -1.012 to 0.972; p = 0.967).

There was no significant change in the statistical results between all 20 questions (diagnostic and therapeutic questions were mixed), and only the ten diagnostic or the ten therapy questions.

### Effect size

The assumed effect size of 0.5 corresponded to a difference in knowledge gain of approximately 1.5 points between group A and group B, taking into account an overall standard deviation (s = 2.973) in knowledge gain between t_0 _and t_1_.

### Intracluster correlation coefficients (ICCs)

The a posteriori calculated ICC for the knowledge test at baseline was 0.054. The a posteriori calculated ICC for the change of knowledge scores was 0.080. The a posteriori calculated ICC for the knowledge at t_1 _with baseline knowledge as covariate was 0.057.

### Secondary outcome: Difference in knowledge gain (t_2_-t_0_)

Study group A (n = 46) and B (n = 51) did not show any statistical significant difference in knowledge gain at t_2 _(2.39 versus 2.00 questions, mean difference: 0.39; CI: -0.83 to 1.61; p = 0.526; T = 0.636). The ANCOVA with QCs as a random effect and the pre-test (t_0_) as covariate achieved a result that can be compared (adjusted mean difference: 0.498; CI: -0.589 to 1.584; p = 0.365).

### Subgroup analyses of users ('per protocol') and non-users of online modules

In study arm A, 47 physicians self-reported in the questionnaire at t_1 _that they had used the online modules ('users' respectively 'per protocol') and 37 indicated that had not used them ('non-users'). Most of the users found the online-modules useful (44 out of 47, 94%). They estimated average activity duration of 83 (15 to 200) minutes. There were no significant differences between the users and non-users in group A regarding gender, age, and pre-test data (t_0_).

A comparison of the 47 users and the 82 participants of group B demonstrated a significant difference in knowledge gain at t_1 _(4.77 questions for 'users' versus 3.60 questions for group B; mean: 1.17; CI: 0.20 to 2.14; p = 0.019; T = 2.38). A cluster analysis with QCs as a random effect and the pre-test (t_0_) as covariate showed a comparable result (adjusted mean difference = 1.115; CI: 0.279 to 1.951; p = 0.009). We also performed a separate analysis to compare the users (n = 47) with the non-users plus group B (n = 119). The result showed a significant effect for the users (adjusted mean difference = 1.845; CI: 0.927-2.764; p < 0.001). In an additional analysis, we found that non-users (n = 37) performed significantly worse than GPs from the group B (n = 82) (adjusted mean difference = -1.529; CI: -2.617 to -0.441; p = 0.009).

Between the 'users' (n = 34) and group B (n = 51) the difference at t_2 _was 2.94 questions for 'users' versus 2.00 questions for group B (mean: 0.94; CI: -0.39 to 2.27; p = 0.164; T = 1.405). A cluster analysis with QCs as a random effect and the pre-test (t_0_) as covariate achieved a similar result (adjusted mean difference = 1.096; CI: -0.10 to 2.292; p = 0.072).

We also performed a separate analysis to compare the users (n = 34) with the non-users plus group B (n = 63). Between them the difference at t_2 _was 2.94 questions for 'users' versus 1.78 questions for 'non users' (group A and group B) (mean: 1.16; CI: -0.095 to 2.422; p = 0.070; T = 1,836).

In contrast, a cluster analysis with QCs as a random effect and the pre-test (t_0_) as covariate showed a significant result (adjusted mean difference = 1.332; CI: 0.222 to 2.442; p = 0.019).

### Outcome of control group

The non-randomised control group (n = 21) also showed an improvement of knowledge, though the knowledge gain at t_2' _(1.48; p = 0.019) was lower compared to the intervention groups at both times.

## Discussion

### Summary of the findings

The purpose of the study was to compare knowledge acquisition about dementia management in GPs between a blended learning approach (online modules in addition to QCs) and QCs ('classical approach') alone [25]. Both educational interventions were based on the dementia guideline of the DEGAM [31]. Our results suggested that the blended learning approach, in which online modules were combined with discussions in QCs, was not superior in knowledge gain to the traditional learning approach in which lectures were combined with discussions in QCs. However, increased knowledge scores were found in both groups, which indicates that there was a positive learning effect with both approaches. A subgroup analysis of the self-reported users of the online modules revealed a benefit of the blended learning approach compared with the traditional lecture approach ('per protocol analysis') as well as a comparison between the users and all other GPs.

### Strengths and limitations of the study

We wanted the WIDA study to have a high external validity and relevance in the context of the GPs environment. As a consequence, we chose the QC setting as the unit of cluster randomisation because more than 50 percent of German GPs are organised in QCs, and QC meetings are also one of the most favoured educational approaches of GPs [29,30,33,40].

The low recruitment rate of clusters (QCs) may appear to compromise the external validity of the study, but this was mostly due to the recruitment procedure. We obtained lists of practising QCs from the responsible medical associations, but only received the information of the specialisation of a QC at the first phone call. The consequence was that many QCs moderators refused to participate at that time because they had had a specialised focus (i.e. diabetes, CAM). This is the reason why the ongoing LISA trial (German acronym for Guideline Implementation Study Asthma) asked the participating GPs to choose their preferred learning style to improve their knowledge on asthma [41]. The personal selection of the learning style might be a reason that the recruitment of GPs was comparatively high [41].

Although participation in QC meetings is mandatory for GPs for some disease management programmes, GPs are not compelled to visit every QC meeting. This may be one reason for the relative high rate of GPs who dropped out during our study. However, low follow-up rates have also been found in other cluster-randomised trials in health service research in primary care settings [42,43].

The main problem of cluster-randomised studies is the risk of selection bias [44], but a comparison of participants' basic data (Table 1) did not find any relevant differences between the blended learning and the 'classical' QCs.

We measured the knowledge gain directly after the second QC meetings (t_1_). This potential advantages the 'classical' approach, because the e-learning intervention took place in the period between t_0 _and t_1 _(Figure 1). In both group A ('blended learning') and group B ('classical' approach) there was case-based group discussion, and this is a potential confounding factor. We could not measure how much this had influenced our results, but we consider that any effect was similar between the two groups [45].

The subgroup analysis of the actual users of the online modules might be biased, because these GPs were probably a more motivated group. Nevertheless, there is a considerable variation in the estimated time for the online modules, from 15 to 200 minutes, which might constitute a problem for implementation. Due to ethical concerns, we did not track users of the online modules and we could not validate the self-reported statements of the 47 GPs ('users') who answered retrospectively that they had used the online modules or the 37 who did not ('non-users'). However, the performed analyses showed that the users not only had a significant knowledge gain compared with group B, but also that the non-users had a significant poorer knowledge gain than group B.

The WIDA-trial had no 'real' randomized control group because we used an additional group to control secular effects and the observation period of this group was shorter. GPs in the control group showed a small but significant knowledge gain that was lower than in the intervention groups at all times. The knowledge gain could be due to the usage of the pocket versions of the dementia guideline that was provided or could be an indicator for a possible ceiling effect, because we used the questionnaire three times in the intervention groups and two times in the control group. The latter seems rather unlikely as no ceiling effect was observed during the IDA trial performed about one year before the WIDA trial [34,35]. It seems improbable that the learning effect by completion of the knowledge test is higher than the one due to the intervention because the study participants received no feedback after the test and the period between the assessment dates was rather long.

Another potential source of bias could be the fact that the GPs received the third (second in the control group) questionnaire by mail, which means that they had had the opportunity to use external material to answer the knowledge questions. However, this risk was the same in all groups.

A major concern of our study might be the primary focus on knowledge. Although the debate about the relationship between competence and performance is important, we did not evaluate performance changes or other outcomes as yet [46-48]. We recognise that educational activities have been shown as only one approach to implement clinical guidelines into practice [2,49,50]. However, educational activities of GPs and health care professionals has been shown to be effective in helping to overcome the taboo on dementia that still exists in Germany [22,51].

### Comparison with existing literature

Recently published studies show that a simple unsolicited distribution of guidelines does not lead to changes in practice [52-56]. For the acceptance and successful implementation of guidelines, a range of selective measures, including CME, CPD, and KT activities, are necessary [23,52-60]. A multifaceted educational program for neurologists was shown to be effective in improving the adoption of a dementia guideline [60], but two other studies showed inadequate implementation of dementia guidelines in general practice [19]. A UK study found that decision support software and practice-based workshops were effective in detecting more people with dementia [23]. However, this study also found that a CD-Rom tutorial was not effective, and this is comparable to findings from a German study [4,23]. Although this trial demonstrated a significant increase in diagnosis rate after intervention, there was no significant improvement in concordance with dementia guidelines on diagnostic and management processes [24]. There still remains doubt about how to effectively implement a dementia guideline, especially in the German general practice context. QCs have been very common during the last decade, and they could be effective in changing practice [30]. However, a QC itself does not guarantee for high quality *per se*. The spectrum of learning activities vary widely, from pharmaceutical-sponsored QCs in restaurants with a high 'entertainment factor' to interactive meetings with substantial and relevant discussions and learning activities. Despite these differences we chose this approach because more than 50 percent of all German GPs have been organized in QCs, and it therefore seemed to be an effective way to reach a relevant number of GPs [29]. During the IDA trial, we offered interested GPs the opportunity to test an e-learning platform [32,34,35]. Most of them had positive feedback, especially those from rural areas.

We also performed a literature review to support our view on the effectiveness of e-learning to improve knowledge and change performance [7,10,27,61,62]. Other authors have also been very optimistic about the use of new technology for CME activities [63,64]. A study by Robson demonstrated an effect of online modules alone on the performance of 45 GPs similar to the findings of Fordis and colleagues [2,10]. Interestingly, both found higher adherence to the recommendations without a gain in knowledge, but Robson asked his participants retrospectively, so there is a high risk of social desirability [2]. A potential bias in the study of Fordis *et al*. is the relatively high reimbursement of their participants [10]. Apart from the pocket version of the guideline and CME credit points, our study abstained from incentives for our participants because we wanted to be as close to reality as possible. We also chose a combination of online modules and group discussion because some studies have identified positive effects of a blend of different learning media, andmore importantly German GPs favour more traditional learning media for their CME activities [11,28,33,40,65,66]. Nevertheless our study suggests that individualised e-learning offerings could be an effective method for transferring relevant knowledge to GPs [67]. Thus, a blended knowledge approach could be one step in a successful implementation strategy addressing the needs or interest of physicians interested in computer-based training, *e.g*., due to the geographic location of their practice [3].

## Summary

Even though our study was not able to identify significant differences in knowledge improvement between the two learning approaches, we are optimistic about the potential of blended learning. First, it may be a regional phenomenon, because barriers to the use of the CME internet activities for German GPs still exists [33]. Second, the minority of the participating GPs who self-reported that they had actually used the online modules showed an increased knowledge gain. Furthermore, 94% of them found the 'e-learning add-on' useful and spent more than one hour with the online modules. Thus, our study depicts that blended learning approaches may provide an effective approach to CME, CPD, and KT in the future. Another positive view is that students are more open to adapt modern technologies and environments into their learning activities [9,12,68]. Future research should address the effectiveness of blended learning arrangements in a framework of a 'CME/CPD/KT' curriculum in contrast to stand-alone solutions [28]. It should also deal with a 'principle of voluntarism' where GPs and other healthcare professionals choose their favourite learning environment [41].

All these approaches should be strictly evaluated, especially if they can change the performance of physicians and/or improve the quality of life of patients [69].

## Ethics Approval

Approval was granted by the Ethics Committee of the Medical Faculty of Witten/Herdecke University (no. 42/2006). The trial was registered in Current Controlled Trials: ISRCTN36550981, and the study protocol has been published [25].

## Competing interests

None of the investigators involved in the study have a conflict of interest. The work was supported by a grant from the Federal Ministry of Education and Research (BMBF) under project number 01GK0512. Any opinions, conclusions, and proposals in the text are those of the authors, and do not necessarily represent the views of the Ministry.

## Authors' contributions

HCV conceived and developed this survey and drafted the manuscript. He collected and collated the data and assisted with statistical analysis. HM performed the statistical analysis and helped to draft the manuscript. TO helped perform the statistical analysis and contributed to draft the manuscript. MB helped design the study. SW assisted in methodological aspects of the survey. MAR helped to design the study and assisted in methodological aspects of the survey. All authors contributed to drafting the manuscript, and read and approved the final manuscript.

## Supplementary Material

Additional file 1 **WIDA-knowledge test**. Questionnaire of the WIDA-trial with 20 multiple choice questions about dementia (in German language). Click here for file
