# Supplementary material for: Knowledge transfer for the management of dementia: a cluster-randomised trial of blended learning in general practice
Source: Implement Sci. 2010 Jan 4;5:1. doi: 10.1186/1748-5908-5-1 (PMC2881109; doi:10.1186/1748-5908-5-1)
Supplement: Additional file 1 — WIDA-knowledge test. Questionnaire of the WIDA-trial with 20 multiple choice questions about dementia (in German language). [file 1748-5908-5-1-S1.PDF]

Qualitätszirkel-Nummer:

Studien-Nummer:

Fragebogen zur Demenz

Positivauswahl
Bei den nachstehenden Aufgaben ist jeweils *eine* Aussage *richtig*. Es geht darum diese zu finden und zu markieren!

1/20		*Hausärzte können den Patienten und ihren Angehörigen nützliche Hinweise zur Gestaltung des häuslichen Umfeldes geben. Welcher der nachstehenden Ratschläge gehört dazu?*
A	☐	Demenzkranke sollten vier Stunden vor dem Zubettgehen die letzte Mahlzeit zu sich nehmen, damit sie nachts weniger unruhig sind.
B	☐	Demenzkranke sollten Musik nur leise hören, damit sie nicht emotional destabilisiert werden.
C	☐	Demenzkranke sollten Kunstlicht vermeiden, damit ihre kognitiven Fähigkeiten nicht überanstrengt werden.
D	☐	Demenzkranke sollten möglichst wenig angesprochen werden, damit sie nicht überfordert werden.
E	☐	Demenzkranke sollten alltägliche Fertigkeiten trainieren, damit sie nicht verloren gehen.

2/20		*Welche Aussage zur Fahrtüchtigkeit bei Demenz ist richtig?*
A	☐	Das Straßenverkehrsamt kann Demenzkranken die Fahrerlaubnis entziehen.
B	☐	Auto fahren ist meist auch für Demenzkranke problemlos möglich.
C	☐	Erst wenn es zu einem Unfall gekommen ist, sollte ein Demenzkranker nicht mehr Auto fahren.
D	☐	Der Hausarzt entscheidet, ob ein Demenzkranker noch Auto fahren darf oder nicht.
E	☐	Bei einer speziellen Fahrprüfung wird entschieden, ob ein Demenzkranker noch Auto fahren darf.

Qualitätszirkel-Nummer:

Studien-Nummer:

3/20		*Welche anamnestische Angabe spricht am wahrscheinlichsten für eine beginnende Demenz?*
A	☐	Der Patient hat Probleme mit seinem Appetit.
B	☐	Der Patient hat Probleme mit Abgeschlagenheit.
C	☐	Der Patient hat neuerdings Probleme mit Angst vor Insekten.
D	☐	Der Patient hat Probleme, seinen Stuhl- und Urinabgang zu kontrollieren.
E	☐	Der Patient hat Probleme, die richtigen Worte zu finden.

4/20		*Was ist ein typisches Frühsymptom einer Demenz?*
A	☐	Distanzlosigkeit gegenüber Fremden
B	☐	Probleme bei der räumlichen Orientierung
C	☐	Störungen des Tag-Nacht-Rhythmus
D	☐	Neigung zu Hebephrenie
E	☐	Gesteigerte Sexualität

5/20		*Wann sollte ein Patient, bei dem eine antidementielle Therapie begonnen wurde, zu einer Verlaufskontrolluntersuchung einbestellt werden? Spätestens nach…*
A	☐	…2 Wochen
B	☐	…1-2 Monaten
C	☐	…3-6 Monaten
D	☐	…7-9 Monaten
E	☐	…10-12 Monaten

6/20		*Welche Aussage zur Epidemiologie der Demenz in Deutschland trifft zu?*
A	☐	Bei ca. ½ Mio. Menschen besteht eine Demenz.
B	☐	Bei ca. 1 Mio. Menschen besteht eine Demenz.
C	☐	Bei ca. 2 Mio. Menschen besteht eine Demenz.
D	☐	Bei ca. 3 Mio. Menschen besteht eine Demenz.
E	☐	Bei ca. 4 Mio. Menschen besteht eine Demenz.

7/20		*Welches Vorgehen wird bei nichtkognitiven Störungen (wie z. B. Aggressivität) vorrangig empfohlen?*
A	☐	Heimeinweisung
B	☐	Therapie mit Cholinesterasehemmern
C	☐	Therapie mit Carbamazepin
D	☐	Therapie mit Benzodiazepinen
E	☐	Nichtmedikamentöse Therapie

8/20		*Wann wird bei einer länger bestehenden Demenz eine neurologische/psychiatrische/geriatrische Konsultation empfohlen? Bei...*
A	☐	... Punktwerten unter 6 im Mini-Mental-Status-Test.
B	☐	… der jährlichen Kontrolle.
C	☐	… zunehmendem sozialem Rückzug des Patienten.
D	☐	... Problemen mit der medikamentösen Therapie.
E	☐	… geplanter Heimeinweisung.

9/20		*Welches Medikament ist zur Behandlung von Schlafstörungen bei Demenzkranken geeignet?*
A	☐	Das Benzodiazepin Lorazepam
B	☐	Das Benzodiazepin Oxazepam
C	☐	Der Benzodiazepin-Agonist Zopiclon
D	☐	Das Neuroleptikum Haloperidol
E	☐	Das trizyklische Antidepressivum Amitriptylin

10/20		*Die Anwendung welcher Substanz(gruppe) kann im Stadium der leichten bis mittleren Alzheimer Demenz sinnvoll sein?*
A	☐	Memantine
B	☐	Ginkgo biloba
C	☐	Vitamin E
D	☐	Cholinesterasehemmer
E	☐	Keine der genannten

Qualitätszirkel-Nummer:

Studien-Nummer:

11/20		*Der mentale Leistungstest DemTect® bildet 5 Bereiche ab, die schon im Frühstadium einer Demenz beeinträchtigt sein können. Welcher der genannten gehört dazu?*
A	☐	Zeitliche Orientierung
B	☐	Emotionale Stabilität
C	☐	Sprachproduktion
D	☐	Neugier
E	☐	Aggressionspotenzial

12/20		*Wenn der Verdacht auf eine Demenz besteht – worauf ist bei der körperlichen Untersuchung besonders zu achten? Auf…*
A	☐	… den Hautturgor
B	☐	… verstärktes Schwitzen
C	☐	… vereinzelte Petechien
D	☐	… eine Landkartenzunge
E	☐	… neurologische Ausfälle

13/20		*Die nachstehenden Erkrankungen können zu dementiellen Symptomen führen. Bei welcher Erkrankung sind diese potenziell reversibel?*
A	☐	Lewy-Body-Demenz
B	☐	Alzheimer-Demenz
C	☐	Normaldruck-Hydrozephalus
D	☐	Amyotrophe Lateralsklerose (ALS)
E	☐	Morbus Pick

14/20		*Die Anwendung welcher Substanz(gruppe) kann im Stadium der mittleren bis schweren Alzheimer Demenz sinnvoll sein?*
A	☐	Memantine
B	☐	Ginkgo biloba
C	☐	Vitamin E
D	☐	Cholinesterasehemmer
E	☐	Keine der genannten

15/20		*Wann wird ein Computertomogramm des Kopfes in der Demenz-Diagnostik empfohlen? Ein Schädel-CT...*
A	☐	... wird empfohlen bei allen unklaren Verläufen.
B	☐	... wird empfohlen vor Heimeinweisungen.
C	☐	... ist verzichtbar, wenn eine Single Photon Emission Computertomographie (SPECT) durchgeführt wird.
D	☐	... ist verzichtbar, wenn ein Elektroenzephalogramm (EEG) durchgeführt wird.
E	☐	…ist verzichtbar, wenn eine Positronen-Emissions-Tomographie (PET) durchgeführt wird.

16/20		*Wann wird bei einem Demenzverdacht auch eine neurologische/psychiatrische/geriatrische Konsultation empfohlen? Bei...*
A	☐	… Komorbidität, z.B. Diabetes mellitus
B	☐	… Patienten- oder Angehörigenwunsch
C	☐	…allen Patienten, die jünger als 65 Jahre sind
D	☐	…allen Patienten, die älter als 65 Jahre sind
E	☐	... allen Patienten mit Demenzverdacht

17/20		*Welche Aussage zur Therapie der Demenz trifft zu?*
A	☐	Marcumar ist Acetylsalicylsäure bei vaskulärer Demenz vorzuziehen.
B	☐	Donepezil ist ein Cholinesterasehemmer, der einmal täglich eingenommen wird.
C	☐	Piracetam verbessert die zerebrale Perfusion und damit die Demenzsymptome.
D	☐	Rivastigmin ist der Cholinesterasehemmer mit dem günstigsten Nebenwirkungsprofil.
E	☐	Gingko-biloba-Präparate verbessern die zerebrale Perfusion und damit die Demenzsymptome.

Qualitätszirkel-Nummer:

Studien-Nummer:

Negativauswahl<br>Bei den nachstehenden Aufgaben ist jeweils *eine* Aussage *falsch*. Es geht darum diese zu finden und zu markieren!

18/20		*Welche Laboruntersuchung wird für ein erstes Screening im Rahmen einer Demenz-Diagnostik nicht empfohlen?*
A	☐	Kalzium
B	☐	Blutbild
C	☐	Glukose
D	☐	TSH
E	☐	LDH

19/20		*Der Mini-Mental-Status-Test wird häufig zur Verlaufsdiagnostik der Demenz benutzt. Was fragt er nicht ab? Die Fähigkeit …*
A	☐	… zur zeitlichen und räumlichen Orientierung
B	☐	… einen Satz zu schreiben
C	☐	… Worte rückwärts zu buchstabieren
D	☐	… ein Telefon zu benutzen
E	☐	… ein überlappendes Fünfeck abzuzeichnen.

20/20		*Was ist kein Risikofaktor für eine Demenz?*
A	☐	Kompensierte Niereninsuffizienz
B	☐	Niedriges Bildungsniveau
C	☐	Nahe Verwandte mit Demenz
D	☐	Erhöhter Homozysteinspiegel
E	☐	Vorangegangener Schlaganfall
